# Drug Repurposing of Verapamil for H1N1 Influenza Virus Infection: A Multi-Target Strategy Revealed by Network Pharmacology and Experimental Validation

**DOI:** 10.3390/ijms27062534

**Published:** 2026-03-10

**Authors:** Yan Cao, Jiajing Wu, Xuena Li, Feifan Qiu, Shuo Wang, Bingshuo Qian, Lingjun Fan, Yueqi Wang, Kun Xue, Junkui Zhang, Beilei Shen, Yuwei Gao

**Affiliations:** 1 Changchun Veterinary Research Institute, Chinese Academy of Agricultural Sciences, State Key Laboratory of Pathogen and Biosecurity, Key Laboratory of Jilin Province for Zoonosis Prevention and Control, Changchun 130122, China; 2 School of Pharmacy, Henan University, Kaifeng 475004, China

**Keywords:** anti-influenza H1N1, verapamil, drug repurposing, molecular dynamics simulation, network pharmacology

## Abstract

Influenza A virus (IAV) infection constitutes a major public health threat. Severe influenza virus infection can induce intense inflammatory responses and lung injury, leading to serious clinical symptoms or even death. The utility of current anti-influenza drugs is often limited by side effects and the emergence of drug-resistant strains. Based on the critical role of L-type voltage-gated calcium channels (L-VGCCs) in influenza virus replication, this study investigates the antiviral activity and mechanism of verapamil, a classic L-type calcium channel antagonist, against H1N1-UI182 virus. Verapamil, an L-type calcium channel blocker, is widely used in the treatment of cardiovascular diseases and has a well-established safety profile. Through molecular dynamics (MD) simulation and network pharmacology analysis, we predicted the stable binding mode of verapamil to the target protein (PDB id: 6JPA) and its potential multi-target network. In vitro, verapamil exhibited antiviral activity against H1N1-UI182 in MDCK cells, enhancing the survival rate of infected cells and reducing viral nucleoprotein (NP) expression. In a lethal H1N1-UI182 infection mouse model, verapamil treatment markedly improved survival rates, alleviated weight loss and lung pathological damage, exhibiting a dose-dependent protective effect. Lung tissue analysis showed that verapamil effectively reduced the lung index and viral load, suppressed the activation of the Nuclear factor kappa B (NF-κB) signaling pathway, and decreased the expression of key inflammatory factors, thereby mitigating the cytokine storm. A comparison of administration regimens indicated that pre-treatment yielded optimal efficacy, suggesting verapamil acts primarily during the early stage of the viral life cycle. This study systematically elucidates that verapamil exerts antiviral and immunomodulatory effects by regulating the NF-κB pathway. Network pharmacology analysis suggested the potential involvement of multiple targets and pathways, including EGFR, SRC, and phospholipase D signaling, providing hypotheses for future mechanistic investigation. This paper supports a drug repurposing strategy against drug-resistant influenza viruses and highlights its significant potential for clinical translation.

## 1. Introduction

Influenza A virus (IAV), an enveloped, segmented, negative-sense RNA virus of the family Orthomyxoviridae, is a major etiological agent of influenza [1]. The Orthomyxoviridae family comprises four genera: influenza A, B, C, and D viruses [2]. Among these, IAV is a primary human respiratory pathogen responsible for seasonal epidemics, causing an estimated 290,000 to 650,000 influenza-associated respiratory deaths annually and posing a substantial global public health burden [3]. Currently, clinical treatments primarily rely on neuraminidase inhibitors such as Oseltamivir and RNA polymerase inhibitors such as Baloxavir (BA). However, the emergence of drug-resistant strains has significantly compromised their efficacy [4,5,6], underscoring the urgent need for novel antiviral candidates [7].

Calcium ions (Ca^2+^) serve as crucial intracellular second messengers, regulating a wide range of cellular processes, including muscle contraction, neurotransmitter release, gene expression, and cell death [8,9]. Ca^2+^ is essential for key stages of the viral life cycle, such as viral entry, viral genome replication, virion maturation, and release [10]. A rise in intracellular Ca^2+^ concentration is a key signal for successful influenza virus infection and replication. IAV infection triggers Ca^2+^ influx into host cells, and elevated cytosolic Ca^2+^ can modulate virus internalization [11,12], The L-type voltage-gated calcium channels (L-VGCCs) have been demonstrated to be directly involved in the influenza virus entry mechanism. Specifically, the influenza virus hemagglutinin can bind to the L-type calcium channel subtype Cav1.2, triggering calcium influx. This, in turn, activates the protease TMPRSS2, facilitating the fusion of the viral envelope with the host cell membrane [13]. Collectively, these findings suggest that L-type calcium channel inhibitors can exert antiviral effects by interfering with this process through the inhibition of Ca^2+^ influx [14,15,16].

Verapamil, a classic L-type calcium channel blocker, has been in clinical use with a well-established safety profile since its approval in 1981. It is commonly employed in the treatment of hypertension [17], diabetes [18], arrhythmias [19], and other cardiovascular diseases [20]. Early research has indicated its antiviral potential; notably, verapamil was first reported in 1984 to inhibit influenza virus replication in MDCK cells [21], However, the specific mechanisms underlying its anti-influenza effects—particularly its impact on the viral life cycle and the association with its anti-inflammatory properties—require further in-depth investigation. Therefore, this study aims to systematically evaluate the antiviral activity of verapamil against influenza viruses through a combination of computational simulations and experimental validation. MD simulations were employed to analyze the dynamic interactions between verapamil and its primary pharmacological target, the L-type calcium channel. Network pharmacology was utilized as a hypothesis-generating tool to systematically predict potential multi-target effects at a systems level, while in vitro and in vivo experiments were conducted to validate key functional outcomes, particularly the modulation of inflammatory pathways.

## 2. Result

### 2.1. In Vitro Antiviral Activity of Verapamil

**Figure 1:**
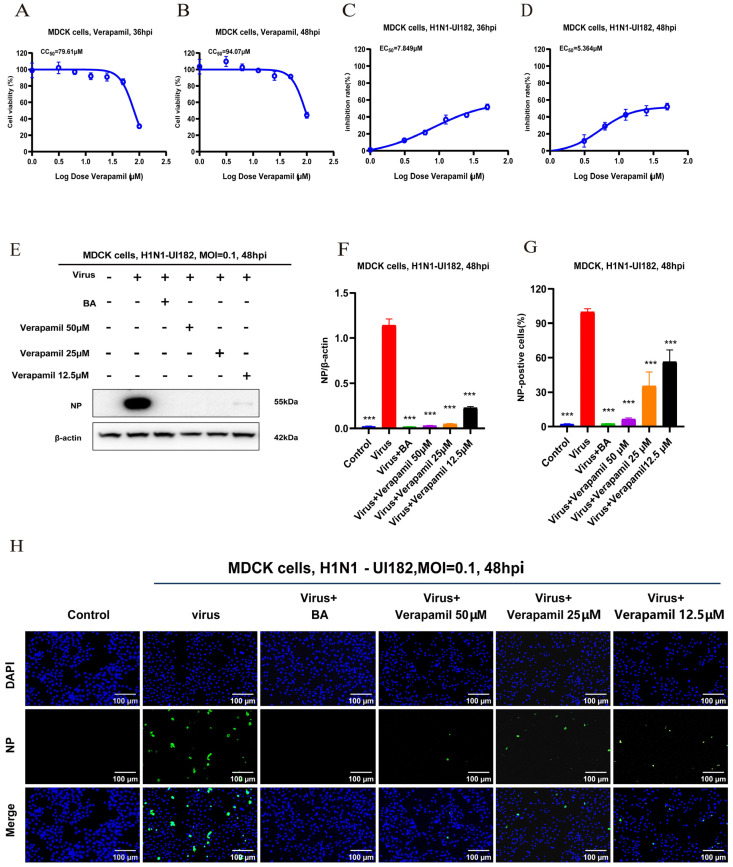
In vitro antiviral activity of verapamil. (**A**,**B**) The CCK-8 assay was used to assess the cytotoxicity of selected drugs on MDCK. (**C**,**D**) The CCK-8 assay was employed to evaluate the therapeutic effects of selected drugs on influenza virus-infected MDCK cells, thereby assessing their potential efficacy. (**E**) Samples collected 48 h post-treatment were analyzed via Western blot imaging to quantify H1N1-UI182 viral nucleoprotein (NP) expression. (**F**) This figure presented the quantified results of Western blot images, showing the average values from three independent experiments compared to the viral control group (±SD) (*n* = 3). *** *p* < 0.001 indicates statistically significant differences. (**G**) Quantitative analysis of immunofluorescence images from Figure (**H**). Results represent the mean of three independent experiments and were compared to the viral control group. (**H**) Immunofluorescence detection of viral nucleoprotein (NP) expression in MDCK cells infected with influenza virus after 48 h of drug incubation. (±SD) (*n* = 3). *** *p* < 0.001 for significant difference.

To evaluate the antiviral activity of verapamil at the cellular level, we first determined its safe concentration range in MDCK. Verapamil was diluted to five concentrations: 1, 12.5, 25, 50, and 100 µM (Figure S1A,B). The results show that verapamil exhibits significant cytotoxicity in MDCK cells at 100 µM. Furthermore, we calculated the half-maximal cytotoxic concentration (CC_50_) of verapamil. In MDCK cells, the CC_50_ values were 79.61 µM at 36 h (Figure 1A) and 94.07 µM at 48 h (Figure 1B). Since the CC_50_ values in both cell lines at different time points were all greater than 50 µM, a concentration of ≤50 µM was selected for all subsequent cellular experiments to ensure safety.

Subsequently, the antiviral activity of verapamil was assessed in H1N1-UI182-infected MDCK cells using five concentration gradients: 3.125, 6.25, 12.5, 25, and 50 µM (Figure S1C,D). Cytopathic effect (CPE) was assessed at 36 and 48 h post-infection. The results demonstrate that verapamil inhibits H1N1 virus replication in a concentration-dependent manner. The half-maximal effective concentration (EC_50_) of verapamil was determined to be 7.849 µM at 36 h and 5.364 µM at 48 h. The SI value was 10.14 at 36 h and 17.54 at 48 h.

In the early stage of infection, the viral nucleoprotein (vNP) localized to the nucleus. During the later stages of infection, it was transported into the cytoplasm and packaged into progeny virions [22]. To further evaluate the inhibitory effect of verapamil on influenza virus viral protein (NP) expression, we conducted additional IF and WB experiments. Drug concentrations of 12.5, 25, and 50 μM were selected, with baloxavir as the positive control. In the IF experiments, NP expression was significantly reduced in the drug-treated groups compared to untreated infected cells (Figure 1G,H). WB results demonstrate that drug treatment significantly suppresses influenza virus NP expression (Figure 1E,F). Collectively, these results indicate that verapamil effectively reduces viral replication and spread within cells. These findings provided a basis for the subsequent experimental investigations.

### 2.2. In Vivo Antiviral Activity of Verapamil

#### 2.2.1. Protective Effect of Verapamil on Survival in Mice with Lethal H1N1 Infection

**Figure 2:**
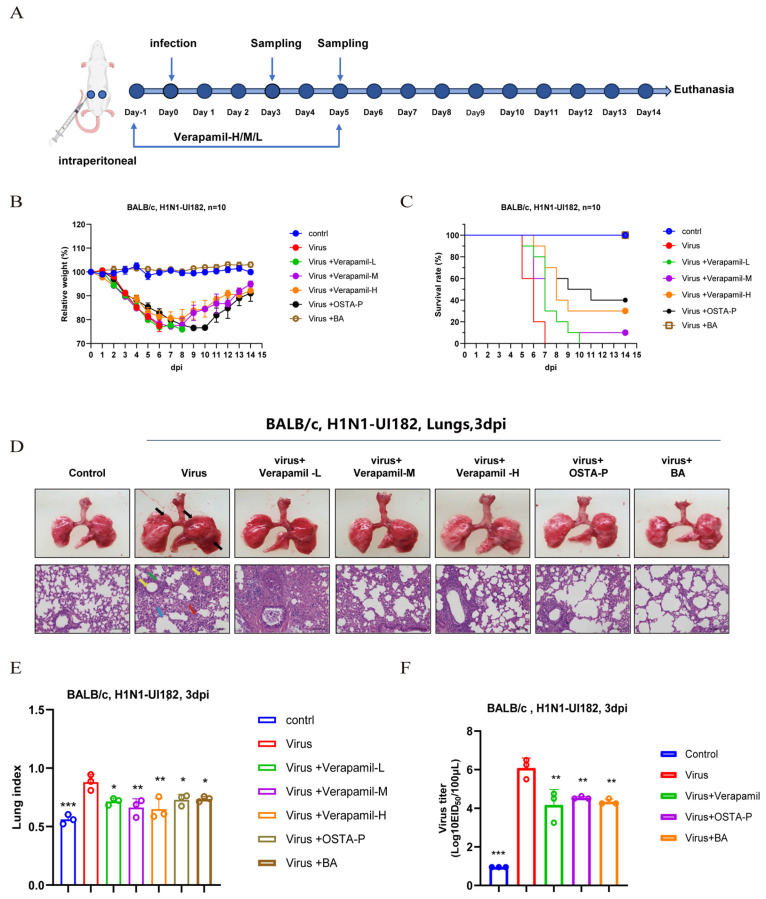
Verapamil protected mice from lethal influenza virus infection. (**A**) Experimental procedure diagram; *n* = 10 mice per group. (**B**) Body weight changes in H1N1-UI182-infected and treated mice. (**C**) Survival rates of H1N1-UI182-infected mice and drug-treated mice. (**D**) After 3 days of infection, we randomly selected 3 mice from each group, euthanized them, and dissected the lungs for H&E staining analysis. (**E**) Changes in lung index in the drug-treated group 3 dpi. (**F**) Changes in viral load in the lungs of mice treated with the drug 3 dpi. *** *p* < 0.001 for significant difference; ** *p* < 0.01 * *p* < 0.05 indicates.

Having demonstrated the antiviral activity of verapamil in vitro, we further evaluated its therapeutic potential in vivo using a lethal H1N1 mouse infection model (Figure 2A). Mice in the virus-infected group began to exhibit sustained weight loss starting from day 2 post-infection, and all succumbed to the infection by day 7. In contrast, treatment with moderate and high doses of verapamil significantly attenuated infection-induced weight loss. Although weight loss in the low-dose verapamil group paralleled that of the virus control, all verapamil-treated groups showed a dose-dependent increase in survival rate (Figure 2B,C). These results indicate that verapamil possesses potent inhibitory activity against H1N1-UI182 and confers significant protection against lethal influenza virus infection in mice.

#### 2.2.2. Verapamil Ameliorates Lung Injury in Mice with Lethal H1N1 Infection

During severe influenza virus infection, the virus typically infected lower respiratory tract cells, causing cellular damage and inflammatory responses that subsequently trigger viral pneumonia. The lung index, defined as the ratio of lung weight to body weight, also increased significantly, reflecting the degree of swelling in lung tissue due to inflammation, edema, or congestion.

We further evaluated the effect of verapamil on lung injury. Compared with the uninfected control group, the virus-infected group showed a significant increase in lung index, indicating severe pulmonary edema and inflammation. Treatment with different doses of verapamil markedly reduced the lung index, with the high-dose verapamil group showing an even lower lung index than the positive control (OSTA-P) group. Gross pathology and hematoxylin and eosin (H&E) staining revealed severe lung lesions in the virus group, which were substantially ameliorated by medium and high doses of verapamil. In contrast, the low-dose group exhibited lesion severity similar to the virus control (Figure 2D,E).

Based on the aforementioned results, the high-dose verapamil group was selected for viral load quantification in mouse lung tissue. The results demonstrate that the viral titer in lung homogenates is significantly reduced at 3 days post-infection (dpi) in the verapamil-treated group (Figure 2F). In summary, verapamil treatment markedly ameliorated lung pathology in infected mice.

#### 2.2.3. Molecular Mechanism Underlying the Anti-H1N1 Activity of Verapamil

**Figure 3:**
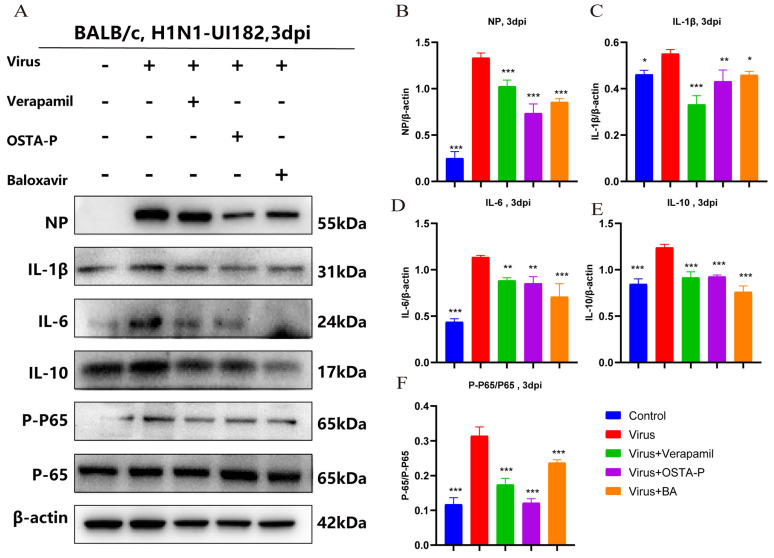
(**A**–**F**) Western blot images show the expression of viral nucleoprotein (NP), IL-1β, IL-6, IL-10, P-P65, and P65. Results represent the mean of three independent experiments and were compared with the viral control group (±SD). *** *p* < 0.001 for significant difference; ** *p* < 0.01 * *p* < 0.05 indicates.

**Figure 4:**
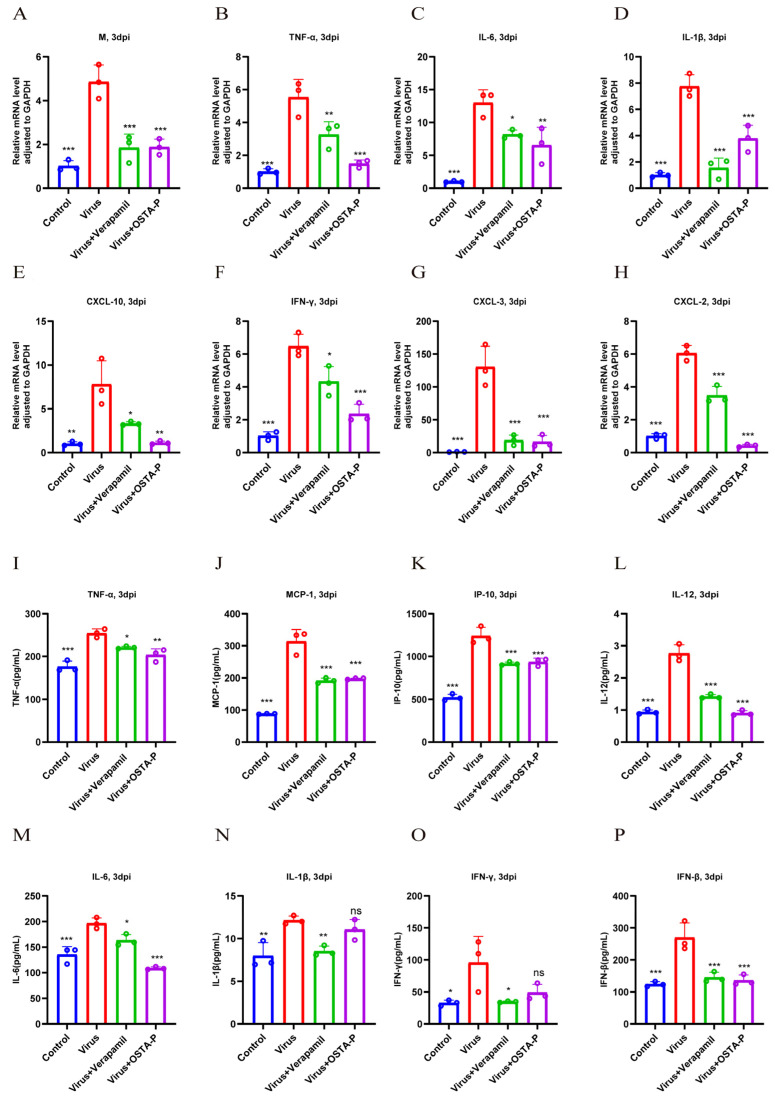
Verapamil improved inflammatory response following viral infection. (**A**–**H**) Expression of cytokines in drug-treated groups was detected using real-time fluorescence quantitative PCR (qPCR). (**I**–**P**) ELISA analysis of cytokine expression after viral infection. Results represent the mean of three independent experiments and are compared with the viral control group (±SD) (*n* = 3). *** *p* < 0.001 for significant difference; ** *p* < 0.01 * *p* < 0.05 indicates. ns, no significant difference.

To investigate the molecular mechanism underlying these protective effects, we evaluated the impact of verapamil on the host inflammatory response. Infection with H1N1-UI182 significantly upregulated the protein expression of key inflammatory cytokines interleukin-6 (IL-6), interleukin-10 (IL-10), interleukin-1 beta (IL-1β), and tumor necrosis factor-alpha (TNF-α) in mouse lung tissue as assessed by Western blot (Figure 3A–F). Consistently, qPCR results demonstrate that a lethal H1N1-UI182 infection substantially upregulates the mRNA expression of cytokines and chemokines, including interferon-gamma (IFN-γ), TNF-α, IL-6, IL-1β, interferon-gamma-induced protein 10 kDa (IP-10), C-X-C motif chemokine ligand 3 (CXCL-3), C-X-C motif chemokine ligand 2 (CXCL-2), and the viral M gene (Figure 4A–H). These inductions were potently suppressed by treatment with verapamil, as well as by the positive controls OSTA-P and baloxavir; in several cases, the inhibitory effect of verapamil was more pronounced than that of the positive controls. Furthermore, ELISA confirmed that verapamil treatment effectively attenuated the increase in levels of IFN-γ, TNF-α, IL-6, IL-1β, interferon-beta (IFN-β), interleukin-12 (IL-12), IP-10, and Monocyte chemoattractant protein-1 (MCP-1) (Figure 4I–P). Collectively, these findings indicate that verapamil effectively mitigates inflammatory responses and reduces viral replication.

The NF-κB pathway plays a crucial role in regulating cytokine and chemokine expression and viral replication during influenza infection [23]. To this end, we examined the phosphorylation status of NF-κB p65. Western blot analysis showed that verapamil treatment reduced virus-induced p65 phosphorylation, consequently inactivating the NF-κB pathway. In summary, our findings reveal that verapamil suppresses influenza-induced inflammation by inhibiting NF-κB signaling.

### 2.3. Protective Effects of Different Administration Regimens of Verapamil in Mice with Lethal H1N1 Infection

**Figure 5:**
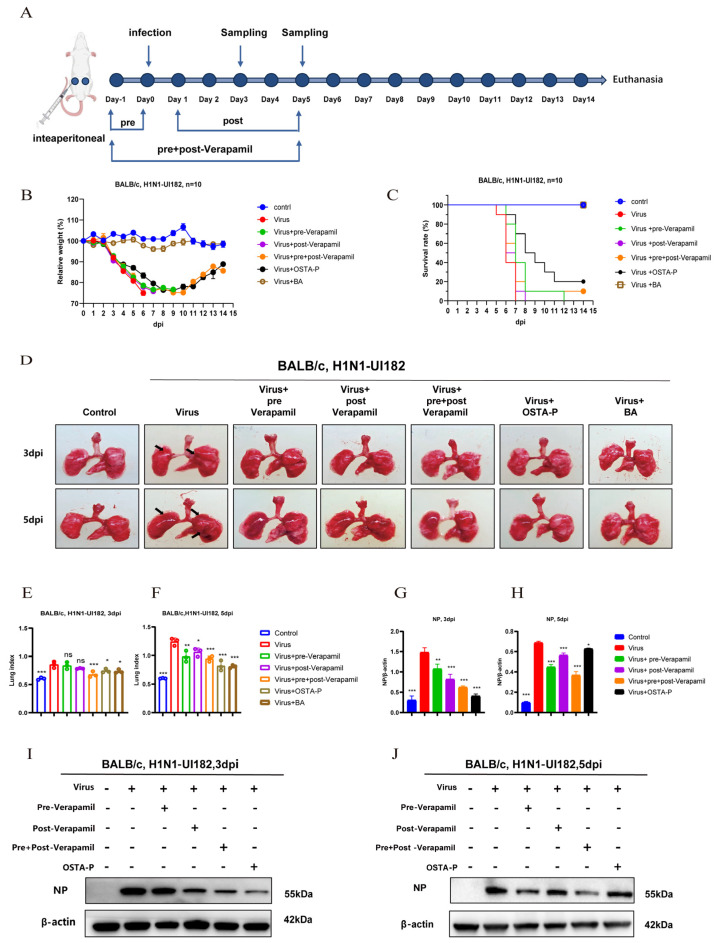
Verapamil protected mice infected with lethal H1N1 virus via different administration routes. (**A**) Experimental procedure diagram; *n* = 10 mice per group. (**B**,**C**) Body weight and survival trends in H1N1-UI182-infected mice and verapamil-treated mice. (**D**) Pulmonary pathological changes in H1N1-UI182-infected mice and drug-treated mice at days 3 and 5 post-infection. (**E**,**F**) Changes in lung index of H1N1-UI182-infected mice treated with verapamil. (**G**–**J**) Western blot images showed viral nucleoprotein (NP) expression in different drug groups; results represent the mean of three independent experiments and are compared with the viral control group (±SD). *** *p* < 0.001 for significant difference; ** *p* < 0.01 * *p* < 0.05 indicates. ns, no significant difference.

To evaluate the therapeutic efficacy of different treatment regimens against H1N1-UI182 infection in BALB/c mice, three administration protocols were compared: verapamil pre-treatment only (pre-Verapamil), verapamil post-treatment only (post-Verapamil), and verapamil combined pre- and post-treatment (pre + post-Verapamil) (Figure 5A). The combined pre- + post-Verapamil regimen conferred superior protection, most effectively mitigating weight loss and improving survival (Figure 5B,C).

Subsequent observation of lung morphology revealed that H1N1-UI182 infection caused significant lung lesions. However, lung pathological damage was markedly ameliorated in the post-Verapamil and pre- + post-Verapamil groups, with the most pronounced improvement observed in the pre- + post-Verapamil groups (Figure 5D). This finding was corroborated by the lung index measurements (Figure 5E,F). Furthermore, Western blot analysis showed that all three treatment groups reduced the protein expression of the viral nucleoprotein (NP) compared to the virus-infected group (Figure 5G–J), with the pre- + post-Verapamil groups again showing the strongest effect.

In summary, a combined administration strategy of pre- and post-infection verapamil treatment demonstrated enhanced efficacy against H1N1 virus infection.

### 2.4. Interaction Analysis and Molecular Dynamics Simulation of Verapamil with the Receptor Protein

**Figure 6:**
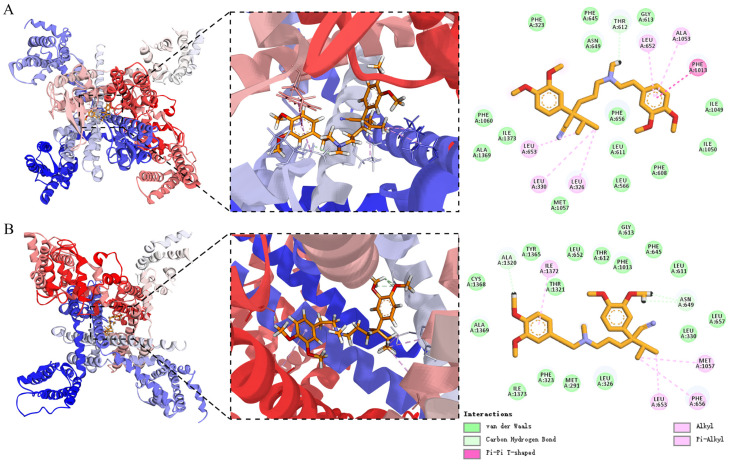
Interaction analysis of verapamil with the receptor protein. (**A**,**B**) The binding conformation of verapamil with the voltage-dependent L-type calcium channel subunit alpha-1S chain.

We visualized and analyzed the interactions between verapamil and its target receptor protein (PDB ID: 6JPA). In 6JPA, verapamil interacts with the Voltage-dependent L-type calcium channel subunit alpha-1S chain in two distinct binding conformations, as shown in Figure 6A,B. In conformation A, verapamil primarily interacts with the calcium channel through π–π stacking, π–alkyl, alkyl, carbon–hydrogen bonds, and van der Waals interactions. Conformation B exhibits similar interaction types; however, it lacks the π–π stacking interaction observed in conformation A. Binding pose scoring was performed for both conformations. The -CDOCKER_INTERACTION_ENERGY value for conformation A was 30.92, whereas that for conformation B was 23.84, indicating that conformation A is the more favorable binding mode. Overall, the complex structure suggests that verapamil binds within the central cavity of the pore domain, thereby blocking ion permeation.

It should be noted that while baloxavir and oseltamivir phosphate were used as positive controls in our in vitro and in vivo experiments, they were not included in the interaction analysis with Cav1.1. This is because these drugs target viral proteins—baloxavir inhibits the influenza polymerase acidic (PA) protein, and oseltamivir targets viral neuraminidase—rather than host calcium channels. Docking them against Cav1.1 would lack biological relevance and could potentially be misleading.

**Figure 7:**
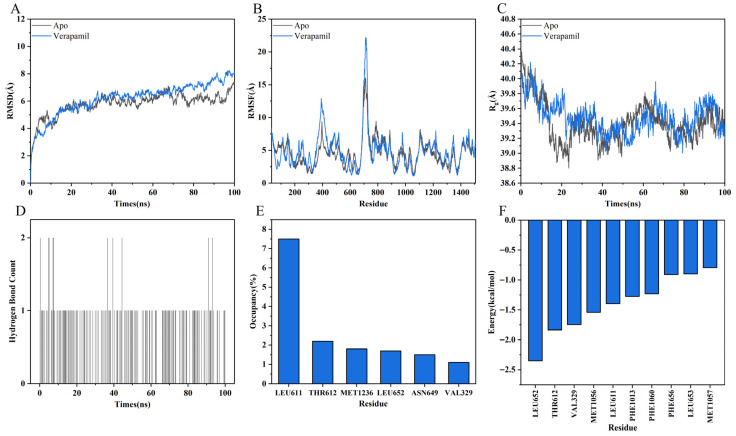
Molecular dynamics simulation analyses. (**A**) RMSD, (**B**) RMSF, and (**C**) R_g_ of the apo protein system (Apo) and the verapamil-bound system (Verapamil). (**D**) Number of hydrogen bonds formed during the simulation. (**E**) Hydrogen bond occupancy of residues with occupancies greater than 1%. (**F**) Top ten residues contributing to the binding free energy.

To capture the dynamic interactions between the ligand and the receptor, a 100 ns MD simulation was performed. As shown in Figure 7A, the backbone root-mean-square deviation (RMSD) values were calculated to evaluate the overall structural stability of the protein in both the Apo and verapamil-bound systems. In both systems, the RMSD values increased during the initial stage of the simulation and gradually reached a plateau in the later phase, indicating that equilibrium was achieved. After approximately 30 ns, the RMSD values of the ligand-bound system were generally slightly higher than those of the Apo system. This increase may reflect ligand-induced conformational rearrangements within the protein.

Residue flexibility was further assessed by analyzing the root-mean-square fluctuation (RMSF), as shown in Figure 7B. Compared with the Apo state, several regions within and adjacent to the binding pocket exhibited reduced RMSF values in the ligand-bound system, particularly around residues 330, 600–650, and 1300. This reduced flexibility is likely attributable to hydrogen bonding, hydrophobic interactions, and electrostatic interactions within the active site. Such restriction of local motions is a mark of stable ligand–protein complex formation. In contrast, certain distal regions showed increased fluctuations upon ligand binding, notably around residues 250, 400, and 700. These observations suggest that ligand binding may induce allosteric effects, propagating dynamic changes to remote structural domains, which are frequently reported in channel proteins.

As shown in Figure 7C, the radius of gyration (Rg) was analyzed to evaluate changes in overall compactness. Throughout the simulation, the Rg values of both systems remained relatively stable, indicating the preservation of global structural integrity. Verapamil binding maintained structural compactness without causing large-scale unfolding or collapse.

Hydrogen bond formation between verapamil and the receptor during the simulation was further analyzed (Figure 7D,E). Verapamil formed up to two concurrent hydrogen bonds with the receptor. The total occupancy of at least one hydrogen bond was 18.1% throughout the simulation. Among the interacting residues, LEU611 showed the highest probability of forming hydrogen bonds with verapamil, with an occupancy of 7.5%.

According to MM-GBSA calculations, verapamil exhibited a strong binding affinity to the receptor, with a predicted binding free energy of −42.52 ± 0.95 kcal/mol. The stability of this complex was primarily driven by key residues including LEU652, THR612, and VAL329 (Figure 7F).

### 2.5. Network Pharmacological Exploration of the Mechanism of Verapamil in Treating Influenza A

#### 2.5.1. Identification of Common Targets Between Verapamil and Influenza A

**Figure 8:**
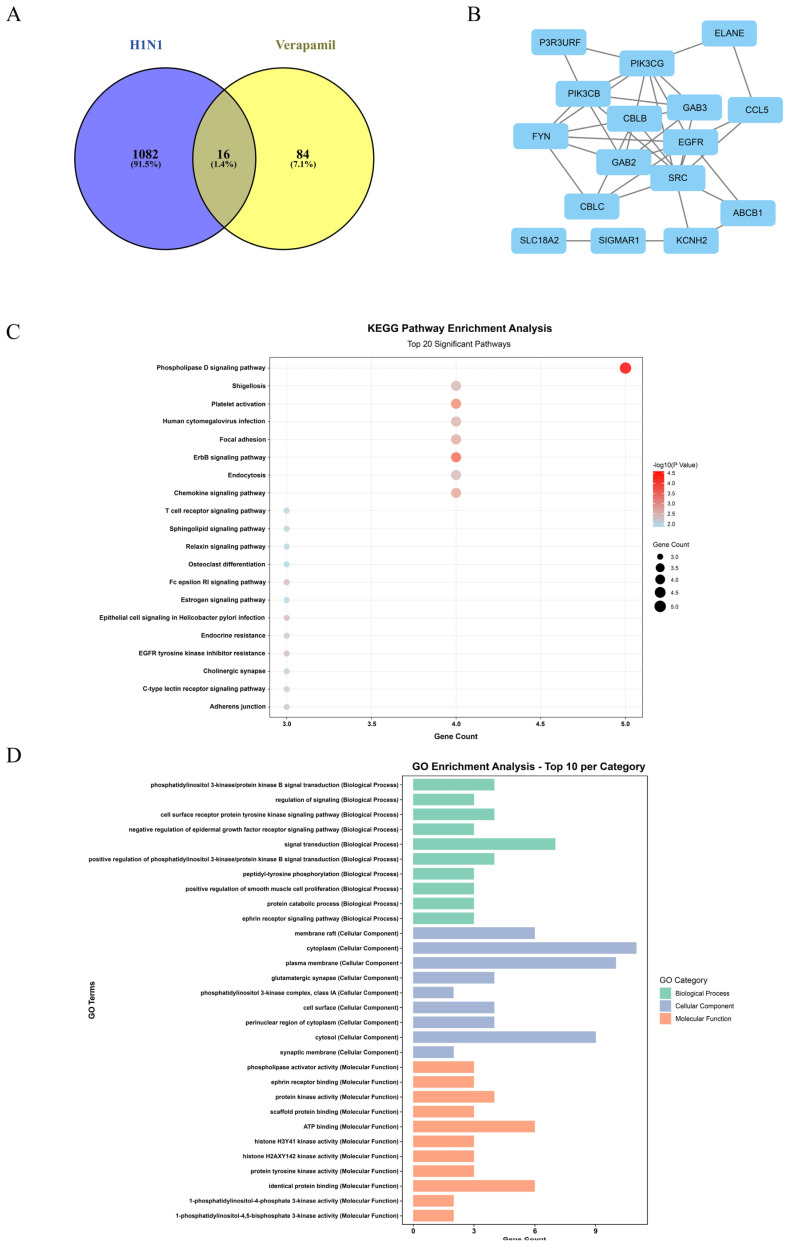
(**A**) Individual and common targets of verapamil and H1N1. (**B**) PPI network constructed to reveal the interactions among the common targets at the protein level. (**C**) KEGG pathway enrichment analysis. (**D**) GO functional enrichment analysis.

To systematically investigate the potential molecular mechanisms of verapamil against H1N1, potential drug targets of verapamil were acquired through database mining and compound structure-based prediction. Concurrently, disease-associated targets related to H1N1 were collected from relevant databases. The Venny 2.1 online tool was employed to perform an interactive mapping between the drug targets and the disease targets, yielding common targets considered potential key targets for verapamil and H1N1. A Venn diagram was generated to visualize this overlap (Figure 8A). As shown, 16 overlapping targets were identified between verapamil and H1N1 suggesting these targets may represent the core through which verapamil exerts its anti-influenza effects. This set of common targets served as the basis for subsequent protein–protein interaction (PPI) network construction and enrichment analyses.

#### 2.5.2. Construction and Analysis of the Protein–Protein Interaction (PPI) Network

To gain deeper insight into the functional relationships among the 16 common targets within a biological system, they were imported into the STRING database to construct a PPI network. The organism was set to “Homo sapiens” with a minimum required interaction confidence score of 0.400. Disconnected nodes were hidden from the network, resulting in a PPI network containing multiple interaction relationships (Figure 8B). This network was then imported into Cytoscape software (version 3.9.1) for visualization and topological analysis. Topological analysis using the CytoNCA plugin identified several targets, including EGFR and SRC, as predicted central hubs within the PPI network based on their high degree, betweenness, and closeness centrality scores. These targets represent potential nodes of interest for future experimental validation.

#### 2.5.3. KEGG Pathway Enrichment Analysis

To elucidate the potential signaling pathways involved in verapamil’s action against H1N1, KEGG pathway enrichment analysis was performed on the 16 common targets, with a significance threshold set at *p*-value < 0.05. The results reveal significant enrichment of the overlapping targets in multiple pathways associated with viral infection, immune inflammation, and cancer (Figure 8C).

The most significantly enriched pathways included the phospholipase D signaling pathway, ErbB signaling pathway, and Platelet activation. It is particularly noteworthy that pathways such as human cytomegalovirus infection and EGFR tyrosine kinase inhibitor resistance were also significantly enriched. These pathways are closely implicated in processes such as viral entry, replication, host immune response, inflammatory cytokine storm, and apoptosis. The analysis suggested that verapamil may exert its therapeutic effects by modulating these keys signaling pathways. These pathways represent predicted mechanisms that may contribute to verapamil’s antiviral effects. In subsequent experiments, we focused on validating the functional outcome of these predicted interactions, particularly the suppression of NF-κB-mediated inflammatory responses.

#### 2.5.4. Gene Ontology (GO) Functional Enrichment Analysis

To further delineate the functional profile of verapamil action at the gene level, GO functional enrichment analysis was conducted on the common targets, covering three categories: Biological Process (BP), Cellular Component (CC), and Molecular Function (MF) (Figure 8D). Biological Process (BP): The targets were significantly enriched in processes such as phosphatidylinositol 3-kinase/protein kinase B signal transduction, regulation of signaling, and cell surface receptor protein tyrosine kinase signaling pathway. These processes are broadly involved in cell proliferation, differentiation, survival, and metabolic regulation, which are frequently hijacked by viruses during infection. CC: The targets were primarily localized to structures like membrane raft, cytoplasm, and plasma membrane. Membrane rafts serve as platforms for various signaling proteins and viral entry receptors, hinting that verapamil might influence virus–host membrane interactions. MF: The targets were mainly associated with functions such as phospholipase activator activity, ephrin receptor binding, and protein kinase activity. These molecular functions are fundamental for transmembrane signal transduction and intracellular cascade reactions.

Collectively, the GO analysis systematically Portrayed the functional context of verapamil’s potential targets across different ontological categories. The results consistently pointed towards a broad modulatory capacity of verapamil on the host cell signaling network, which may constitute an important functional basis for its anti-H1N1 activity.

#### 2.5.5. Integration of Network Pharmacology Predictions with Experimental Findings

To establish a direct correlation between the computational predictions and our experimental data, we mapped the key pathways identified by KEGG enrichment back to our in vitro and in vivo results. The network pharmacology analysis highlighted the phospholipase D and ErbB signaling pathways, which are known upstream regulators of the NF-κB inflammatory cascade. This prediction provided a mechanistic hypothesis that was subsequently tested and confirmed by our experimental work. As shown in Figure 3 and Figure 4, verapamil treatment significantly inhibited the activation of the NF-κB pathway (reduced p-P65 levels) and suppressed the expression of downstream pro-inflammatory cytokines (IL-6, TNF-α, IL-1β) at both the transcriptional and protein levels. This convergence validates that the multi-target network predicted in silico functionally translates to the modulation of critical inflammatory pathways observed in our infection models.

## 3. Discussion

Influenza viruses cause annual epidemics and occasional pandemics of respiratory infections, resulting in a broad spectrum of clinical disease severity in humans [24,25,26]. Consequently, preventing and treating influenza virus infection remains a significant global public health challenge. Numerous studies have indicated that host Ca^2+^ channels are crucial for viral replication and release, positioning them as potential therapeutic antiviral targets [27]. It is noteworthy that cells cannot synthesize or catabolize Ca^2+^; its homeostasis is governed by the complex interplay of Ca^2+^ handling mechanisms, including channels, pumps, and exchangers [28]. One of the cellular signaling cascades most frequently dysregulated during viral infection is cellular calcium (Ca^2+^) dynamics [29,30]. Furthermore, multiple stages of the influenza virus life cycle, from entry to release, are dependent on calcium ions [11]. Therefore, calcium channel inhibitors have garnered considerable attention for their potential application in treating influenza virus infections.

Verapamil, as a prototypical calcium channel inhibitor, can exert antiviral effects at multiple stages of the viral infection cycle. This study comprehensively investigated the anti-influenza potential of verapamil through integrated in vitro and in vivo approaches. Our findings demonstrate that verapamil exhibits potent antiviral activity in cellular models, significantly enhancing the viability of infected cells and reducing viral nucleoprotein (NP) expression. In the mouse model, verapamil treatment significantly improved survival rates and mitigated infection-induced weight loss. MD simulations were employed for dynamic analyses to further investigate the interactions between verapamil and the receptor protein. Network pharmacology analysis revealed the potential multi-target characteristics of verapamil’s anti-influenza effects at the systems level. It predicted that verapamil may regulate key targets such as EGFR and SRC, thereby influencing multiple pathways closely associated with viral infection and immune inflammation—including the phospholipase D signaling pathway and the ErbB signaling pathway. This aligns with our experimental observations of NF-κB pathway inhibition and the downregulation of inflammatory factors. Therefore, we propose that verapamil represents a promising candidate for targeted therapy, offering a viable strategy to address current limitations in antiviral treatment.

However, it is important to note that the CC_50_ values (74.99–94.07 μM) are relatively close to the highest antiviral concentration tested (50 μM). While all antiviral assays included parallel viability controls confirming that the observed effects at ≤50 μM were not attributable to drug-induced cytotoxicity, this proximity highlights the need for careful dose optimization in future in vivo studies and clinical applications to maximize efficacy while minimizing potential toxicity. And, to fully establish the concentration–response relationship, the activity threshold of verapamil below the tested concentrations (e.g., 3.125–6.25 μM) warrants further characterization in future studies. Furthermore, the inclusion of baloxavir as a positive control was based on its distinct mechanism of action targeting the PA endonuclease, which inhibits viral mRNA transcription at an early post-entry stage. This differs from oseltamivir, which targets viral release. By including both classes of antivirals, we aimed to comprehensively evaluate verapamil’s antiviral efficacy relative to drugs with different mechanisms and stages of action.

Inflammation-mediated lung injury represents a major cause of morbidity and mortality in intensive care unit patients [31,32]. The NF-κB signaling pathway is a classical pro-inflammatory signaling cascade. Upon activation, this pathway promotes the expression of interleukin-1 (IL-1), tumor necrosis factor-alpha (TNF-α), and other pro-inflammatory genes, including cytokines, chemokines, and adhesion molecules [33,34,35].

Our findings demonstrate that verapamil reduces H1N1 infection-induced phosphorylation of p65 and decreases the mRNA and protein levels of multiple cytokines, including TNF-α, IL-1β, IL-6, and IL-10. This establishes a coherent mechanism: verapamil attenuates NF-κB activation, thereby inhibiting inflammatory cytokine production, alleviating the cytokine storm, mitigating lung pathology, and ultimately reducing mortality.

Network pharmacology served as a hypothesis-generating tool, predicting that verapamil’s anti-influenza activity may involve a multi-target network including hubs such as EGFR and SRC, and pathways such as phospholipase D and ErbB signaling. While these specific targets were not directly validated at the molecular level in the present study, they are known to converge on downstream inflammatory cascades, particularly the NF-κB pathway. Our experimental data confirm that verapamil treatment leads to the inhibition of p65 phosphorylation and reduced expression of NF-κB-driven cytokines (IL-6, TNF-α, IL-1β). Thus, the network pharmacology predictions are supported by our experimental findings at the level of shared downstream functional outcomes. This interpretation aligns with verapamil’s established pharmacology as a calcium channel blocker, where NF-κB suppression represents a downstream consequence rather than a direct drug–target interaction.

In our in vivo experiments, a time-of-addition approach was adopted. we hypothesize that achieving elevated concentrations of verapamil prior to viral exposure inhibits viral entry, thereby establishing an “antiviral state” in host cells and more effectively blocking early stages of viral replication. Continuous administration post-infection serves to both sustain the suppression of viral replication and control the subsequent dysregulated inflammatory response. The combined pre- and post-treatment regimen integrates these effects, achieving a dual strategy of “blocking viral entry” and “suppressing subsequent viral assault,” which culminates in maximized therapeutic efficacy.

While the present study demonstrates the antiviral efficacy of verapamil against H1N1-UI182 influenza virus, the precise stage of the viral life cycle affected by this compound remains to be fully elucidated. Based on the role of calcium channels in viral entry and fusion, future studies in our laboratory will focus on time-of-addition experiments and viral binding/internalization assays to definitively establish whether verapamil exerts its antiviral effect through inhibition of early viral entry events.

As a drug approved in 1981, verapamil has established a comprehensive human safety profile, with well-characterized and clinically manageable common side effects such as hypotension and bradycardia. Repurposing such a drug can significantly shorten development timelines and reduce costs, which is crucial for pandemic preparedness. Based on its unique mechanism of action—targeting host calcium channels—and the multi-target regulatory potential suggested by network pharmacology, verapamil may remain effective against viral strains resistant to current neuraminidase inhibitors (e.g., oseltamivir) or polymerase acidic (PA) inhibitors (e.g., baloxavir), representing a significant potential advantage. Although these findings indicate that verapamil may have broader antiviral potential, this study has certain limitations. The specific stage of viral entry targeted by verapamil (attachment, internalization, or fusion) requires further exploration. The antiviral activity of verapamil was assessed in this study primarily via NP protein expression and against a single viral strain (H1N1-UI182). Future investigations should incorporate direct quantification of viral RNA and other viral proteins (HA, M1, NA) to validate these findings. Moreover, testing against a broader range of influenza strains—including other IAV subtypes, influenza B virus, and drug-resistant variants—is necessary to establish the generalizability of verapamil’s antiviral efficacy. Furthermore, Future studies are needed to thoroughly investigate its effects on the early stages of viral infection. This would help elucidate its broad-spectrum antiviral potential and provide a solid preclinical foundation for conducting clinical trials on verapamil for the treatment of severe influenza.

## 4. Materials and Methods

### 4.1. Cell Culture, Reagents, Virus, and Mice

Fetal bovine serum (FBS), a 10,000 U/mL penicillin–streptomycin solution, and Dulbecco modified Eagle culture Medium (DMEM) were purchased from Thermo Fisher Scientific Corporation (Shanghai, China). Cell Counting Kit-8 (CCK-8) (Beyotime, Shanghai, China, C0039, CCK-8) and dimethyl sulfoxide (DMSO) were purchased from Sigma-Aldrich (Shanghai, China). The virus used in this study was the mouse-adapted strain (H1N1-UI182) of the influenza A H1N1 2009 virus (A/Vinig/01/2009 (H1N1)). The H1N1 virus was inoculated into MDCK cells. Both the virus and the cell lines were preserved at the Institute of Changchun Veterinary Research, Chinese Academy of Agricultural Sciences (Changchun, China). MDCK cells were cultured in DMEM supplemented with 5% fetal bovine serum (FBS, Gibco, Lot No. 10438026), respectively, along with 100 IU/mL penicillin and 100 µg/mL streptomycin.

Verapamil in this article (CAS No.: 52-53-9) was purchased from MCE (South Brunswick, NJ, USA). According to the manufacturer’s instructions, the drug was dissolved DMSO and subsequently diluted in DMEM.

### 4.2. Cytotoxicity Test and In Vitro Antiviral Activity Determination

Cells were seeded in 96-well plates and cultured in DMEM containing 5% fetal bovine serum and 1% (*w*/*v*) penicillin–streptomycin (37 °C, 5% CO_2_). When the cell confluence reached 80%, viral inoculation and cytotoxicity assays were performed. Virus-infected cells and uninfected cells were simultaneously treated with working solutions at various concentrations. Positive control wells (virus only) and negative control wells (without virus) were established for each plate. After 36 and 48 h of culture, cell viability was determined using the Cell Counting Kit-8 (Beyotime, Shanghai, China, C0039, CCK-8) to assess viral infection and cytotoxicity. The absorbance at 450 nm for each well was measured using an ELISA plate reader. A nonlinear regression curve fitting analysis of cell viability versus the log10-transformed drug concentration was performed using GraphPad Prism 8 to calculate EC_50_ for infection and CC_50_ for cell viability.

### 4.3. Immunofluorescence Staining (IF)

Cells were seeded in a 24-well plate. When cell confluence reached 50%, they were infected with H1N1/UI182 (MOI = 0.5). At 48 h post-infection, the medium was discarded, and the cells were washed 2–3 times with phosphate-buffered saline (PBS, P1020, Solabio, Beijing, China). The cells were then fixed with 4% paraformaldehyde (PFA) for 20 min, followed by permeabilization with 0.5% Triton X-100 (P0096, Beyotime, China) for 20 min. After blocking with 2% bovine serum albumin (BSA, 9048-46-8, Sigma, Burlington, MA, USA) for 1 h and one wash, the cells were incubated with the primary antibody (GeneTex, San Antonio, TX, USA, GTX125989, 1:1000) at room temperature for 1 h, followed by three washes. Subsequently, the cells were incubated with the secondary antibody (Bioss, Beijing, China, bs-0295G-FITC, 1:1000) for 1 h. Nuclei were stained with Hoechst 33,258 (Termo Fisher Scientific, Waltham, MA, USA, H3569,1 μg/mL) for 10 min, followed by one wash. Finally, the cells were observed and recorded using a fluorescence microscope (Carl Zeiss, Oberkochen, Germany).

### 4.4. In Vivo Experiments

#### 4.4.1. In Vivo Inhibitory Effects of Verapamil on Virus

Female BALB/c mice (6–8 weeks old, 15–20 g) were purchased from Liaoning Changsheng Biotechnology Co., Ltd., Benxi, China. After one week of adaptive feeding, the mice were randomly divided into two cohorts (A and B). Both cohorts underwent identical experimental conditions. Cohort A was used to monitor body weight changes and survival rates, while Cohort B was designated for analyzing lung pathological changes.

Each cohort was further randomly divided into seven groups (*n* = 10 per group): control, virus-infected (Virus), high-dose verapamil treatment (Verapamil-H), medium-dose verapamil treatment (Verapamil-M), low-dose verapamil treatment (Verapamil-L), oseltamivir phosphate (OSTA-P) treatment, and baloxavir (BA) treatment groups (BA, a cap-dependent endonuclease inhibitor that targets the PA subunit of the influenza virus RNA polymerase complex, was used as a positive control representing an early-acting antiviral. OSTA-P, a neuraminidase inhibitor that blocks viral release, was included as a comparator representing a late-acting antiviral.) All groups except the control were intranasally inoculated with IAV virus. Pre-treatment was administered to the Verapamil-H, Verapamil-M, and Verapamil-L groups via intraperitoneal injection (i.p.) [27] (i.p.) for two days prior to virus infection, at doses of 6.25 mg/kg/day, 3.125 mg/kg/day, and 1.5625 mg/kg/day, respectively. Treatment continued for 5 dpi, once daily. For the positive control groups, baloxavir (5 mg/kg/day) was administered subcutaneously once daily, and oseltamivir phosphate (25 mg/kg/day) was given orally twice daily with an 8 h interval, starting after virus infection. The virus-infected model group received daily intraperitoneal injections of 0.9% saline post-infection. Mice in Cohort A were monitored daily for 14 consecutive days to record clinical signs, including body weight loss, survival status, and disease progression. For Cohort B, tissue samples were collected specifically at 3 dpi. Lung tissues were harvested for the determination of lung index, measurement of viral titer, assessment of local histopathological changes, and analysis of differences in inflammatory factor expression.

#### 4.4.2. Protective Effects of Verapamil Administered at Different Time Points in Mice with Lethal H1N1 Infection

Healthy 6–8-week-old female mice (BALB/c strain, weighing 15–20 g) from Liaoning Changsheng Biotechnology Co., Ltd., Benxi, China, were acclimated for one week and randomly divided into Groups A and B. Group A monitored body weight changes and survival rates, while Group B assessed pulmonary pathological alterations. Both groups maintained identical experimental conditions. Each group was further randomized into seven subgroups of ten mice each. Experimental groups included: control, model (Virus), verapamil prophylactic treatment (pre), verapamil therapeutic treatment (post), verapamil combined pre- and post-treatment (pre + post), oseltamivir (OSTA-P) treatment, and baloxavir. Except for the control group, all other groups received intranasal inoculation with IAV virus. Prior to viral infection, the verapamil combined prevention and treatment group (pre + post, 6.25 mg/kg/d, i.p.) received pre-treatment for 2 days, followed by continued dosing for 5 dpi, administered once daily. The verapamil prophylactic treatment group (pre, 6.25 mg/kg/d, i.p.) received pre-treatment for 2 days at once daily prior to infection and no treatment post-infection. The verapamil treatment group (pre + post, 6.25 mg/kg/d, i.p.) continued treatment for 5 days at once daily post-infection. The positive control group received baloxavir (5 mg/kg/d, s.c.) once daily after viral infection; OSTA-P (25 mg/kg/d, i.g.) twice daily at 8 h intervals; the model group received 0.9% saline (i.p.) after viral infection. Group A was continuously monitored for 14 days to observe weight loss, survival, and clinical signs of disease in mice across all groups. Group B collected tissue samples on days 3 and 5 post-infection (dpi), including mouse lung tissue, to measure lung index, lung tissue viral titer, local histopathological changes, and investigate differences in inflammatory factor expression.

#### 4.4.3. RNA Isolation and Quantitative RT-qPCR

Weigh equal amounts of tissue from each group, then extract total RNA from each tissue sample using the HiPure Universal RNA extraction Kit (R4130-03, Magen, Guangzhou, China). Extracted RNA was measured using a nanophotometer (Emplen, NP80, Munich, Germany), followed by normalization of RNA concentration. RNA of equal concentration and volume was reverse transcribed into cDNA using the PrimeScript™ RT Kit (Takara, RR047A, Kyoto, Japan) according to the manufacturer’s instructions. Subsequently, gene expression was quantified using an RT-qPCR system (Bio-Rad, Hercules, CA, USA) with GAPDH as a loading control. Primer sequences are shown in Table 1.

#### 4.4.4. ELASA

Wash lung tissue with pre-chilled PBS (0.01 M, pH = 7.4) to remove residual blood, weigh the tissue, and then mince it. Grind the minced tissue with an equal volume of PBS (1:9 weight-to-volume ratio) at 40 Hz for 6 min. Finally, centrifuge the lung tissue at 2–8 °C and 12,000 rpm for 10–15 min, then collect the supernatant for detection. Proceed according to the protocol for the Mouse Cell and Chemokine ELISA Detection Kit provided by Elabscience Biotechnology Co., Ltd., Wuhan, China.

#### 4.4.5. EID_50_ Detection

To determine the EID_50_ using 10-day-old SPF chicken embryos, mouse lung tissue stored at −80 °C was thawed on ice. Four sterile steel balls (3 mm) and DMEM medium containing diamantin were added to centrifuge tubes, followed by homogenization at 40 Hz for 6 min. Centrifuge at 4 °C and 12,000 rpm. Dilute the supernatant sequentially from 10^−1^ to 10^−8^ in 10-fold dilutions (3 replicates per gradient). Inject 100 virus solution into 3 10-day-old SPF chicken embryos per concentration. Seal each embryo with paraffin. After 48 h, 1% chicken red blood cells (RBCs) washed in water served as the indicator system. For each egg, 50 µL of allantoic fluid was mixed with 50 µL of 1% chicken RBC. The mixture was incubated at room temperature for 15 min, and the hemagglutination results in the chicken embryos were measured. Virus titers were calculated using the Reed–Muench method, with results expressed as log10 EID_50_.

#### 4.4.6. Pathological Analysis

On day 3 post-viral infection, three mice from each group were euthanized for tissue collection. Tissues were fixed at room temperature with 4% PFA) and subjected to H&E staining. Fixed tissues were dehydrated with ethanol and paraffin-embedded. Processed tissue sections were stained with H&E solution (Thermo, Shanghai, China), dried, and scanned using a digital microscope (Olympus, Tokyo, Japan). Endogenous peroxidase activity was blocked by incubating sections at room temperature with 0.3% H_2_O_2_ in methanol for 20 min. Following antigen retrieval, tissue sections were blocked with 5% goat serum at room temperature for 20 min, then incubated overnight at 4 °C with anti-influenza A virus antibody (vNP, Abcam, Shanghai, China). After washing, tissue slides were incubated with biotinylated secondary antibody (Abcam, China) at room temperature for 1 h. Slides were stained with DAB (3,3′-diaminobenzidine, Thermo, China) and counterstained with hematoxylin. Finally, slides were evaluated under a microscope (Olympus, Japan).

#### 4.4.7. Western Blot (WB)

Tissue proteins were extracted using RIPA lysis buffer (P0013B, Beyotime, China). For each sample, 10–15 µg of protein was loaded onto an SDS-polyacrylamide gel and separated by electrophoresis. Protein concentration was determined using a BCA protein assay kit (P0010S, Beyotime, China). After denaturation, the separated proteins were transferred onto a PVDF membrane and blocked with 5% BSA. Subsequently, the membrane was incubated overnight at 4 °C with the corresponding primary antibody. Following incubation with a secondary antibody for 2 h, the membrane was washed three times for 10 min each with TBST. Finally, proteins were detected using a chemiluminescence imaging system (Tanon, Shanghai, China), and grayscale analysis and quantification were performed using ImageJ software (version 1.54p) (NIH, Bethesda, MD, USA), with β-actin serving as the internal reference protein.

The following antibodies were used: NP (Abcam, ab104870, 1:1000), β-actin (Abcam, ab6276, 1:500), IL-1β (Proteintech, Rosemont, IL, USA, 16806-1-AP), IL-10 (CST, Danvers, MA, USA, D13A11), IL-6 (CST, D5W4V), IFN-β (CST, D2J1D), p65 (CST, D14E12), p-p65 (CST, 93H1), goat anti-rabbit IgG (Beyotime, A0208, 1:1000), and goat anti-mouse IgG (Beyotime, A0216, 1:1000).

### 4.5. Interaction Analysis and Molecular Dynamics Simulation

To elucidate the interaction mechanism between verapamil and the target receptor, PyMOL 2.3.0 [36] and Discovery Studio was employed to analyze and visualize the protein–ligand interactions. In addition, the two conformations of verapamil present in the original PDB structure were evaluated using the Score Ligand Poses module. The conformation with the higher score was subsequently selected for MD simulations.

Prior to the MD simulations, the alpha-1S chain was modeled using AlphaFold3 to reconstruct the missing residues in the intermediate region. The modeled alpha-1S chain was then aligned to its original position in the PDB structure, yielding a RMSD of 1.27 Å, indicating good structural agreement and reliability of the constructed model.

During the MD simulation setup, the system was subjected to a two-step energy minimization process. First, 2000 steps of steepest descent minimization were performed, followed by an additional 2000 steps using the conjugate gradient method. During minimization, positional restraints with a force constant of 2.0 kcal/mol/Å^2^ were imposed on all protein and ligand atoms. Subsequently, the system was slowly heated from 0.1 K to 300 K over a 50 ps simulation in the NVT ensemble, employing a Langevin thermostat with a collision frequency (γ_ln) of 2.0 ps^−1^. The same positional restraints were maintained during the heating phase. After reaching 300 K, a 50 ps density equilibration was carried out in the NPT ensemble under continued restraints. This was followed by a 500 ps equilibration in the NPT ensemble at 300 K and 1 atm without any restraints. Throughout all stages of the simulations, bonds involving hydrogen atoms were constrained using the SHAKE algorithm, enabling the use of a 2 fs integration time step. Finally, a 100 ns production molecular dynamics simulation was conducted in the NPT ensemble under periodic boundary conditions. Temperature and pressure were controlled using the Langevin thermostat and the Berendsen barostat (pressure relaxation time τ_p = 5.0 ps), respectively. A nonbonded interaction cutoff of 8.0 Å was applied, and long-range electrostatic interactions were treated with the Particle Mesh Ewald (PME) method.

### 4.6. Network Pharmacology Analysis

This study employs network pharmacology methods to elucidate the potential multi-target mechanisms of candidate drugs. First the SwissTargetPrediction database (http://www.swisstargetprediction.ch/, accessed on 14 December 2025) was utilized to identify potential protein targets for the candidate drugs, with a probability threshold set at >0.1. Predicted targets were standardized to official gene symbols. Subsequently, the human gene database GeneCards was used to collect targets associated with H1N1. The intersection of drug-predicted targets and disease-related targets was taken to obtain potential therapeutic targets. The STRING database (version 11.5) was utilized to construct a protein–protein interaction network for these shared targets, with a minimum interaction confidence score set at 0.4 (medium confidence). Visualization and analysis of the resulting network were performed using Cytoscape software (version 3.9.1). Its CytoHubba plugin was employed to calculate key topological parameters including degree, betweenness centrality, and closeness centrality to identify core targets. Finally, functional enrichment analysis of the common targets was conducted using the DAVID bioinformatics database (version 6.8) [37,38].

### 4.7. In Vivo Experimental Design

Sample size justification: Sample sizes were chosen based on established practices in influenza antiviral research [39,40,41] and the 3Rs principle to minimize animal use while ensuring reliable results.

Randomization: Mice were randomly allocated to treatment groups using a computer-generated randomization list prepared by an independent researcher. Cages were arranged in a randomized block design to minimize positional effects.

Blinding: All procedures and assessments were blinded. Drug solutions were coded by an independent researcher. Body weight and survival were monitored daily by blinded staff. Lung tissue analyses were performed by investigators unaware of group assignments. Unblinding occurred only after data collection.

### 4.8. Statistical Analysis

All data were statistically analyzed using GraphPad Prism 8.0 and are presented as mean ± standard error of the mean (SEM). One-way ANOVA or Student’s *t* test was used to determine significant differences. Differences were considered statistically significant at *p* < 0.05 Symbols indicate significance levels: * *p* < 0.05, ** *p* < 0.01, and *** *p* < 0.001. ns denotes no statistically significant difference.

## 5. Conclusions

This study provides a solid foundation for the antiviral effects of verapamil, further confirming it as an effective candidate drug against H1N1-UI182 virus. Through integrated in vitro and in vivo experiments, we demonstrate that verapamil exerts a dual effect: inhibiting viral replication and alleviating inflammatory responses via the modulation of the NF-κB pathway.

Network pharmacology analysis predicted a multi-target interaction network potentially involving hubs such as EGFR and SRC, as well as pathways including phospholipase D and ErbB signaling. While these specific targets require direct experimental validation in future studies, our experimental data confirm that a key functional outcome of verapamil treatment is the suppression of the NF-κB pathway and its downstream inflammatory cytokines (IL-6, TNF-α, IL-1β)—a central node that converges with the predicted network. This partial alignment between computational predictions and experimental findings provides a foundation for more targeted mechanistic investigations in the future.

The host-targeted characteristics of verapamil offer innovative approaches to addressing current antiviral drug resistance challenges. Its well-established safety profile and potential multi-target effects warrant further clinical translation studies with a broader panel of influenza virus strains, particularly for severe influenza infections where inflammation plays a critical pathogenic role.

## Figures and Tables

**Table 1:** The sequence of primer for RT-qPCR.

Gene Name	Primer Sequence (5′ to 3′)
GAPDH	F: 5′-ACATCAAGAAGGTGGTGAAGCA-3′
	R: 5′-CTTGACAAAGTGGTCGTTGAGG-3′
M	F: 5′-5CACACACGTCTCCGGGAGCAAAAGCAGGTAG-3′
	R5′-CACACACGTCTCCTATTAGTAGAAACAAGGTAGTTTTT-3′
IFN-γ	F: 5′-AGCCAAATCGTCTCCTTCTACTTC-3′
	R: 5′-TGCACCTTGTTGCTGCTGTT-3′
TNF-α	F: 5′-AGCCCTGGTATGAACCCATC-3′
	R: 5′-GGAATCGGCAAAGTCAAGGT-3′
IL-1β	F:5′-TCATCGTGGCAGTGGAAAAG-3′
	R: 5′-GGGAAGCAAGGGTCTCAGGT-3′
IL-6	F: 5′-AGTTGCCTTCTTGGGACTGATG-3′
	R: 5′-GGGAGTGGTATCCTCTGTGAAGTCT-3′
CXCL-10	F: 5′-CAGCAGTCCGCAGTATAAACAGT-3′
	R: 5′-GCCAAGTACCTAACGCTCACC-3′
CXCL-3	F: 5′-GCTCAACATCATGAAGGTCTCC-3′
	R: 5′-TGCCGGTTTCTCTTAGTCAGG-3′
CXCL-2	F: 5′-CTCAAGAACATCCAAAGTGTG-3′
	R: 5′-ATTCTTGAGTGTGGCTATGAC-3′

## Data Availability

Dataset available on request from the authors.

## Supplementary Materials

The following supporting information can be downloaded at: https://www.mdpi.com/article/10.3390/ijms27062534/s1. Figure S1. A–D, presented with linear scaling for clarity, are now available in the Supplementary Materials.

## Abbreviations

OSTA-P	Oseltamivir phosphate
BA	Baloxavir
CC50	Half maximal cytotoxic concentration
EC50	Half maximal effective concentration
EID50	50% Egg Infectious Dose
H&E	Hematoxylin and Eosin
DPI	Days post-infection
IV	Influenza Virus
IAV	Influenza A Virus
IL-6	Interleukin 6
IL-10	Interleukin 10
IFN-β	Interferon β
IL-1β	Interleukin-1 β
TNF-α	Tumor necrosis factor-α
IFN-γ	Interferon-γ
IP-10	Interferon-gamma-induced protein 10 kDa
CXCL3	C-X-C motif chemokine ligand 3
CXCL2	C-X-C motif chemokine ligand 2
MCP-1	Monocyte chemoattractant protein-1
NF-κB	Nuclear factor kappa B
MDCK	Madin–Darby Canine Kidney
CC	Cellular Component
MF	Molecular Function
M	Matrix protein
NP	Nucleoprotein
PA	Polymerase acidic
PFA	Paraformaldehyde
RBCs	Red Blood Cells
RT-qPCR	Real-Time quantitative fluorescent PCR
WB	Western Blot
DMSO	Dimethyl Sulfoxide
FBS	Fetal bovine serum
DMEM	Dulbecco modified Eagle culture Medium
CCK-8	Cell Counting Kit -8
BSA	Bovine Serum Albumin
BA	Balanced Accuracy
IF	Immunofluorescence staining
