# Supplementary material for: Drug Repurposing of Verapamil for H1N1 Influenza Virus Infection: A Multi-Target Strategy Revealed by Network Pharmacology and Experimental Validation
Source: Int J Mol Sci. 2026 Mar 10;27(6):2534. doi: 10.3390/ijms27062534 (PMC13027264; doi:10.3390/ijms27062534)
Supplement: Supplementary file 1 [file ijms-27-02534-s001.zip › ijms-4176047-supplementary.pdf]

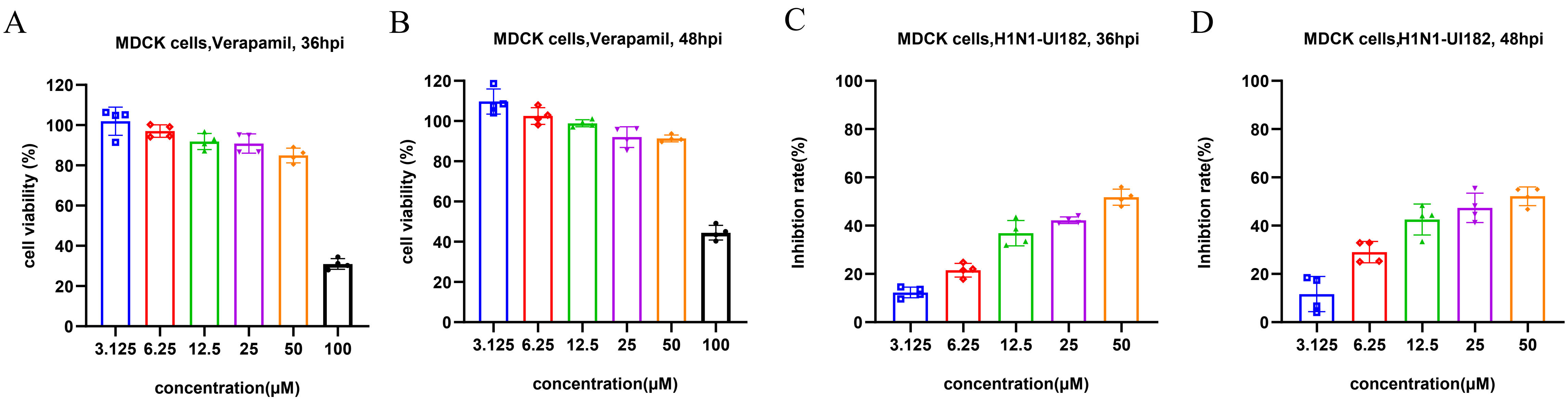

Figure S1. A–D, presented with linear scaling for clarity, are now available in the Supplementary Materials.
